# Sex-Specific Outcomes in Patients Undergoing Mitral Valve Transcatheter Edge-to-Edge Repair: The REPAIR Study

**DOI:** 10.1016/j.jacadv.2026.102782

**Published:** 2026-05-13

**Authors:** Philipp von Stein, Florian Schindhelm, Felix Rudolph, Paula Sagmeister, Jean Marc Haurand, Jannik Jobst, Matthias Gröger, Lukas Stolz, Carl Schulz, Christoph Alexander Mues, Donika Mustafa, Isabel A. Hoerbrand, Atsushi Sugiura, Charlotte Wolff, Philipp Lurz, Jennifer von Stein, Guido Ascione, Henning Guthoff, Stephan Baldus, Wolfgang Rottbauer, Muhammed Gerçek, Roman Pfister, Tienush Rassaf, Marcel Weber, Mathias Konstandin, Helge Möllmann, Niklas Schofer, Jörg Hausleiter, Mirjam Kessler, Bernhard Unsöld, Patrick Horn, Tobias Kister, Volker Rudolph, Amir Abbas Mahabadi, Juan F. Granada, Victor Mauri, the REPAIR Investigators, Ralph-Stephan von Bardeleben, Cecilia Ennin, Kai Peter Friedrichs, Christina Grothusen, Malte Kelm, Georg Nickenig, Kerstin Piayda, Tobias Ruf, Leonhard Moritz Schneider, Holger Thiele, Laila Widmann

**Affiliations:** a Department of Cardiology, Heart Center, Faculty of Medicine, University of Cologne, Cologne, Germany; b Cardiovascular Research Foundation, New York, New York, USA; c Center for Cardiovascular Medicine ABCD, Aachen – Bonn – Cologne – Düsseldorf, Germany; d Department of Cardiology and Vascular Medicine, West German Heart and Vascular Center, University Hospital Essen, University of Duisburg Essen, Essen, Germany; e Clinic for General and Interventional Cardiology/Angiology, Herz-und Diabeteszentrum NRW, Ruhr-Universität Bochum, Bad Oeynhausen, Germany; f Herz-und Diabeteszentrum Nordrhein-Westfalen, Med. Fakultät OWL (Universität Bielefeld), Bad Oeynhausen, Germany; g Department of Internal Medicine/Cardiology, Heart Center Leipzig at University of Leipzig and Leipzig Heart Science, Leipzig, Germany; h Department of Cardiology, Pulmonology and Vascular Medicine, University Hospital Düsseldorf, Medical Faculty of the Heinrich Heine University Düsseldorf, Düsseldorf, Germany; i Department of Cardiology and Angiology, Justus-Liebig-University Giessen, Giessen, Germany; j Department of Internal Medicine II, Ulm University Heart Center, Ulm, Germany; k Medizinische Klinik und Poliklinik I, Ludwig-Maximilians-Universität München, Munich, Germany; l Munich Heart Alliance, Partner Site German Center for Cardiovascular Disease (DZHK), Munich, Germany; m Department of Cardiology, University Heart & Vascular Center Hamburg, University Medical Center Hamburg-Eppendorf, Hamburg, Germany; n Medical Clinic I, Department of Cardiology, St. Johannes Hospital, Dortmund, Germany; o Department of Internal Medicine III, Division of Cardiology, University Hospital Heidelberg, Ruprecht-Karls University Heidelberg, Heidelberg, Germany; p Department of Internal Medicine II, Heart Center Bonn, University Hospital Bonn, Bonn, Germany; q Department of Cardiology, University Medical Center of the Johannes Gutenberg-University Mainz, Mainz, Germany

**Keywords:** M-TEER, mean gradient, mitral regurgitation, real-world, REPAIR, sex-specific

## Abstract

**Background:**

Mitral valve transcatheter edge-to-edge repair (M-TEER) is an established treatment for patients with mitral regurgitation (MR) at prohibitive surgical risk. Sex-specific M-TEER outcomes are mainly derived from small, historic, MR etiology–specific, or MitraClip-treated cohorts.

**Objectives:**

The objective of the study was to evaluate sex-specific outcomes in patients undergoing PASCAL M-TEER.

**Methods:**

REgistry of PAscal for mItral Regurgitation is an investigator-initiated, multicenter M-TEER registry. Outcomes included Mitral Valve Academic Research Consortium-defined technical success, optimal result at discharge (residual MR ≤ 1+ and mean transmitral pressure gradient <5 mm Hg), and 1-year all-cause mortality.

**Results:**

Among 2,601 patients, 1,150 (44.2%) were females and 1,451 (55.8%) were males. MR etiology distribution was similar between sexes (primary: 33.5% vs 32.4%; secondary: 48.9% vs 52.1%; mixed: 17.6% vs 15.6%; *P* = 0.219). Technical success was achieved in 97.0% vs 97.7% (*P* = 0.395). An optimal result was achieved more often in males (55.7% vs 65.9%; *P* < 0.001), driven by higher postprocedural gradients in females (mean transmitral valve pressure gradient ≥5 mm Hg: 24.0% vs 14.0%; *P* < 0.001), whereas residual mild or less MR was similar (71.3% vs 74.6%; *P* = 0.066). One-year mortality was 11.0% (95% CI: 8.7%-13.2%) vs 12.1% (95% CI: 10.1%-14.1%; *P* = 0.266). Residual mild or less MR was independently associated with lower 1-year mortality (adjusted HR: 0.57; 95% CI: 0.38-0.84; *P* = 0.005), whereas gradients <5 mm Hg were not (adjusted HR: 0.63; 95% CI: 0.40-1.01; *P* = 0.056).

**Conclusions:**

PASCAL M-TEER resulted in similar technical success rates and 1-year mortality across sexes, despite females achieving an optimal result less frequently, due to higher postprocedural gradients. MR reduction to mild or less was the primary determinant of improved survival, consistent across sexes.

Mitral regurgitation (MR) is a prevalent valvular heart disease in older adults and is associated with impaired quality of life, heart failure hospitalizations, and increased mortality.^1^^,^^2^ Mitral valve transcatheter edge-to-edge repair (M-TEER) has emerged as an established treatment for symptomatic, moderate-to-severe, or severe MR in patients at an increased surgical risk.^3^

Despite a similar distribution of MR across sexes and advances in transcatheter heart valve therapies, females remain under-represented in cardiovascular device trials and registries, and continue to experience disparities in referral patterns, baseline risk profiles, and procedural characteristics.^4^, ^5^, ^6^, ^7^, ^8^, ^9^ Accordingly, females accounted for only approximately one-third of participants in key M-TEER studies, including 36% in EVEREST II (Endovascular Valve Edge-to-Edge Repair Study), 36% in COAPT (Cardiovascular Outcomes Assessment of the MitraClip Percutaneous Therapy for Heart Failure Patients with Functional Mitral Regurgitation), and 35% in CLASP IID.^6^^,^^9^^,^^10^

Prior studies evaluating sex-specific outcomes after M-TEER have demonstrated similar clinical outcomes between females and males, despite substantial differences in baseline characteristics.^6^^,^^7^^,^^9^^,^^11^, ^12^, ^13^, ^14^, ^15^ However, these studies were largely limited by small sample sizes, inclusion of historic populations, MR etiology–specific reporting, and their exclusive or predominant use of the MitraClip system.^6^^,^^7^^,^^9^^,^^11^, ^12^, ^13^, ^14^, ^15^ Therefore, robust, sex-specific data for newer-generation M-TEER systems in contemporary, real-world practice remain limited.

The PASCAL (Edwards Lifesciences) system represents an M-TEER technology with distinct design features, including independent leaflet clasping and broad maneuverability of the device, which may be particularly relevant in anatomically diverse patient populations.^16^ Yet, large-scale real-world evidence addressing sex-specific procedural performance, echocardiographic results, and clinical outcomes with this system is sparse.

Accordingly, this study aimed to evaluate sex-specific baseline characteristics as well as procedural and 1-year clinical outcomes in the largest real-world cohort of patients undergoing M-TEER with the PASCAL system, the REgistry of PAscal for mItral Regurgitation (REPAIR) (DRKS00033959).

## Methods

### Study design and patient population

The design and methodology of the REPAIR study have been detailed previously.^17^ In brief, REPAIR is an ongoing, investigator-initiated, observational, multicenter registry enrolling MR patients treated with the PASCAL M-TEER system between 2019 and 2024 at 14 centers. All patients were evaluated by local interdisciplinary heart teams for M-TEER eligibility based on clinical characteristics, anatomical suitability, and optimization of maximum tolerated guideline-directed medical therapy (GDMT).

All M-TEER procedures were performed using the PASCAL system, of which the implantation procedure has been previously described.^16^ In the absence of established consensus criteria, the choice between the PASCAL and MitraClip systems was left to the discretion of local physicians. Echocardiographic assessments were conducted by experienced clinicians at each center in accordance with established guidelines.^18^^,^^19^ MR severity was evaluated preintervention, at discharge, short-term follow-up, and latest available follow-up using a multiparametric approach, with severity classified as none/trace (0), mild (1+), mild-to-moderate (2+), moderate-to-severe (3+), and severe (4+).^18^^,^^19^

MR etiology was site reported as primary MR, secondary MR (SMR), or mixed MR. Three-dimensional (3D) mitral valve orifice area (MVOA) was obtained intraprocedurally from 3D transesophageal echocardiography using multiplanar reconstruction or direct 3D measurement, according to local practice. Tricuspid regurgitation (TR) was also graded using a multiparametric approach with severity classified as none/trace, mild, moderate, and severe.^18^^,^^19^

The study was conducted in accordance with the principles of the Declaration of Helsinki, received ethical approval from the appropriate Institutional Review Boards, and was registered in the German Clinical Trials Register (DRKS00033959).

### Study definitions

For this analysis, patients were stratified by sex. An optimal result was defined as residual MR ≤1+ and a mean transmitral valve pressure gradient (MPG) of <5 mm Hg.^20^ For descriptive analyses of GDMT in patients with SMR, patients were additionally stratified according to left ventricular ejection fraction (LVEF) into heart failure with reduced (HFrEF) (≤40%), mildly reduced (HFmrEF) (41%-49%), and preserved ejection fraction (HFpEF) (≥50%).^21^

### Study outcomes

The primary endpoint was achieving an optimal result at discharge. Secondary endpoints included the individual components of an optimal result, Mitral Valve Academic Research Consortium (MVARC)-defined technical success, 1-year all-cause mortality, the composite of 1-year all-cause mortality or heart failure hospitalization, 1-year reintervention rate, and the rate of achieving NYHA functional class ≤II.^20^

### Statistical analysis

Data were summarized as mean ± SD, median (Q1–Q3), or frequencies (n [%]). Between-group comparisons were performed using the chi-square test, Fisher exact, or Mann-Whitney U-test, as appropriate. Survival was assessed using the Kaplan-Meier method and compared between groups with the log-rank test, considering time to first event, and estimates are reported with 95% CIs. Time-to-event outcomes were further analyzed using Cox proportional hazards regression models and were estimated as mixed-effects with random intercept for center. Proportional hazards were verified using Schoenfeld residuals. Multivariable Cox regression models included optimal result and sex or residual MR ≤1+, MPG <5 mm Hg, and sex. Fully adjusted models included the same parameters including age, NYHA functional class, diabetes mellitus, coronary artery disease, previous myocardial infarction, previous cardiac surgery, estimated glomerular filtration rate, LVEF, and TR severity. Multiplicative interaction terms were tested individually by including cross-product terms in the model. Effect estimates are reported as HRs with 95% CIs. Two-sided *P* values <0.05 were considered statistically significant and were not adjusted for multiplicity. All statistical analyses were conducted using R (version 4.4.3, R Foundation for Statistical Computing).

## Results

### Baseline characteristics

Among the 2,601 patients who underwent M-TEER in the REPAIR study, 1,150 (44.2%) were females and 1,451 (55.8%) were males. The median follow-up duration was 449 days (Q1-Q3: 352-781 days) and did not differ between sexes.

Detailed baseline demographic and clinical characteristics are shown in Table 1. Compared to males, females were slightly older (median 81 years vs 80 years; *P* < 0.001), had lower N-terminal pro-B-type natriuretic peptide levels (median 2,453 pg/mL vs 2,787 pg/mL; *P* = 0.005), and had a higher European System for Cardiac Operative Risk Evaluation II (EuroSCORE II) score (median 4.9% vs 4.4%; *P* = 0.007). Most comorbidities were less prevalent in females than in males, including diabetes mellitus (20.0% vs 25.8%; *P* < 0.001), coronary artery disease (49.1% vs 67.1%; *P* < 0.001), previous myocardial infarction (13.1% vs 20.9%; *P* < 0.001), and previous cardiac surgery (11.9% vs 24.5%; *P* < 0.001). With respect to symptoms, females more frequently presented in NYHA functional class III (74.2% vs 68.7%; *P* = 0.002) and less frequently in class IV (12.0% vs 15.1%; *P* = 0.026).

**Table 1:** Baseline Characteristics

	Females (n = 1,150)	Males (n = 1,451)	*P* Value
Age, y	81.0 (76.4-84.3)	79.8 (72.8-83.9)	**<0.001**
Body mass index, kg/m^2^	24.7 (22.3-28.3) (n = 1,148)	25.2 (23.4-28.3) (n = 1,450)	**<0.001**
Body surface area, m^2^	1.7 (1.6-1.8) (n = 1,122)	2.0 (1.8-2.1) (n = 1,427)	**<0.001**
NYHA functional class			**0.015**
I	6/1,149 (0.5)	13/1,449 (0.9)	0.378
II	152/1,149 (13.2)	222/1,449 (15.3)	0.146
III	853/1,149 (74.2)	995/1,449 (68.7)	**0.002**
IV	138/1,149 (12.0)	219/1,449 (15.1)	**0.026**
NYHA functional class ≥III	991/1,149 (86.2)	1,214/1,449 (83.8)	0.091
NT-proBNP, pg/mL	2,453 (1,139-4,960) (n = 855)	2,787 (1,305-6,049) (n = 1,070)	**0.005**
EuroSCORE II, %	4.9 (3.2-7.6)	4.4 (2.7-8.2)	**0.007**
Comorbidities			
Arterial hypertension	973/1,146 (84.9)	1,190/1,446 (82.3)	0.085
Diabetes mellitus	230/1,150 (20.0)	374/1,450 (25.8)	**<0.001**
Coronary artery disease	565/1,150 (49.1)	974/1,451 (67.1)	**<0.001**
Previous myocardial infarction	151/1,149 (13.1)	303/1,451 (20.9)	**<0.001**
Previous cardiac surgery	137/1,150 (11.9)	355/1,451 (24.5)	**<0.001**
Previous stroke/TIA	112/1,091 (10.3)	178/1,381 (12.9)	0.051
CRT/ICD/PM	177/1,150 (15.4)	419/1,451 (28.9)	**<0.001**
Atrial fibrillation	818/1,150 (71.1)	1,036/1,451 (71.4)	0.915
Chronic lung disease	262/1,150 (22.8)	365/1,451 (25.2)	0.174
Renal function			
eGFR, mL/min	43.7 (33.0-58.1) (n = 1,148)	50.9 (37.8-69.6) (n = 1,442)	**<0.001**
eGFR <60 mL/min	892/1,148 (77.7%)	911/1,442 (63.2%)	**<0.001**
On dialysis	19/1,069 (1.8%)	36/1,328 (2.7%)	0.168
Medication			
ACEi, ARB, or ARNI	704/1,001 (70.3%)	950/1,288 (73.8%)	0.077
ARNI	109/784 (13.9%)	289/990 (29.2%)	**<0.001**
Beta-blocker	859/1,001 (85.8%)	1,059/1,288 (82.2%)	**0.024**
MRA	405/1,001 (40.5%)	592/1,288 (46.0%)	**0.010**
SGLT2 inhibitor	189/982 (19.2%)	369/1,259 (29.3%)	**<0.001**
Loop diuretic	844/1,001 (84.3%)	1,089/1,288 (84.5%)	0.924

Values are n/N (%) or median (Q1-Q3). **Bold** values indicate statistical significance.

ACEi = angiotensin-converting enzyme inhibitor; ARB = angiotensin receptor blocker; ARNI = angiotensin receptor neprilysin inhibitor; CRT = cardiac resynchronization therapy; eGFR = estimated glomerular filtration rate (calculated using the Cockcroft–Gault equation); EuroSCORE II = European System for Cardiac Operative Risk Evaluation II; ICD = implantable cardioverter-defibrillator; MRA = mineralocorticoid receptor antagonist; NT-proBNP = N-terminal pro–B-type natriuretic peptide; PM = pacemaker; SGLT2 = sodium-glucose cotransporter-2; TIA = transient ischemic attack.

Female patients were less frequently treated with GDMT, including angiotensin receptor neprilysin inhibitors (ARNIs), mineralocorticoid receptor antagonists (MRAs), and sodium-glucose cotransporter-2 inhibitors (SGLT2i). In contrast, beta-blocker use was more common among females, whereas overall treatment with renin-angiotensin system inhibitors was similarly distributed between sexes. At least triple GDMT was administered in 35.6% (356/1,001) of females and 43.3% (559/1,288) of males (*P* < 0.001). Quadruple therapy was applied in 8.8% (86/982) of females compared to 14.6% (184/1,257) of males (*P* < 0.001).

Among patients with SMR, similar sex-based differences were observed. LVEF-stratified analyses revealed sex-based differences across heart failure categories. In HFrEF, females less frequently received ARNIs and SGLT2i, whereas rates of MRA use and at least triple or quadruple GDMT were similar between sexes. In HFmrEF, lower ARNI use in females persisted, whereas other differences were less pronounced. In HFpEF, GDMT rates were largely similar between sexes (Supplemental Table 1).

Baseline echocardiographic characteristics are summarized in Table 2. MR etiology was similarly distributed between sexes (Central Illustration). Females had higher LVEF and smaller left ventricular and left atrial dimensions. The MPG was higher in females, and, in the subset with available measurements, 3D-MVOA was smaller (median 4.2 cm^2^ vs 5.0 cm^2^; *P* < 0.001).

**Table 2:** Baseline Echocardiographic Characteristics

	Females (n = 1,150)	Males (n = 1,451)	*P* Value
Left atrium			
LA volume index, mL/m^2^	56 (43-74) (n = 907)	60 (47-81) (n = 1,112)	**<0.001**
Left ventricle			
LVEF, %	55 (44-60) (n = 1,046)	45 (31-55) (n = 1,345)	**<0.001**
LVEDD, mm	51 (46-57) (n = 890)	58 (52-65) (n = 1,167)	**<0.001**
LVESD, mm	36 (31-44) (n = 683)	45 (36-55) (n = 901)	**<0.001**
LVEDV index, mL/m^2^	59 (45-76) (n = 745)	77 (59-101) (n = 962)	**<0.001**
Mitral valve			
MR Etiology			0.219
PMR	383/1,143 (33.5)	465/1,437 (32.4)	0.565
SMR	559/1,143 (48.9)	748/1,437 (52.1)	0.122
MMR	201/1,143 (17.6)	224/1,437 (15.6)	0.192
MPG, mm Hg	2.0 (1.1-3.0) (n = 930)	2.0 (1.0-2.5) (n = 1,172)	**<0.001**
3D-MVOA, cm^2^	4.2 (3.5-5.2) (n = 522)	5.0 (4.2-6.3) (n = 667)	**<0.001**
VC width, mm	7.0 (6.0-8.5) (n = 757)	7.8 (6.0-9.0) (n = 974)	**<0.001**
EROA, mm^2^	30.0 (22.0-42.0) (n = 945)	39.0 (27.0-50.0) (n = 1,189)	**<0.001**
Regurgitant volume, mL	47.0 (35.0-63.0) (n = 799)	52.0 (38.0-69.0) (n = 1,027)	**<0.001**
MR severity			**0.003**
0+	0/1,148 (0.0)	0/1,449 (0.0)	
1+	0/1,148 (0.0)	0/1,449 (0.0)	
2+	26/1,148 (2.3)	14/1,449 (1.0)	**0.012**
3+	677/1,148 (59.0)	805/1,449 (55.6)	0.088
4+	445/1,148 (38.8)	630/1,449 (43.5)	**0.017**
Right ventricle			
TR severity			**0.019**
None/trace	29/1,032 (2.8)	44/1,266 (3.5)	0.432
Mild	335/1,032 (32.5)	484/1,266 (38.2)	**0.005**
Moderate	384/1,032 (37.2)	423/1,266 (33.4)	0.064
Severe	284/1,032 (27.5)	315/1,266 (24.9)	0.166
TR ≥ moderate	668/1,032 (64.7)	738/1,266 (58.3)	**0.002**
TAPSE, mm	19.0 (15.4-22.0) (n = 966)	18.0 (14.9-21.0) (n = 1,242)	**<0.001**
PASP, mm Hg	44.0 (34.9-55.0) (n = 1,024)	45.0 (35.0-55.0) (n = 1,267)	0.471

Values are n (%) or median (Q1-Q3). **Bold** values indicate statistical significance.

3D = 3-dimensional; EROA = effective regurgitant orifice area; LA = left atrium; LVEF = left ventricular ejection fraction; LVEDD = left ventricular end diastolic diameter; LVESD = left ventricular end systolic diameter; LVEDV = left ventricular end diastolic volume; MMR = mixed MR; MR = mitral regurgitation; MPG = mean mitral valve pressure gradient; MVOA = mitral valve orifice area; PASP = pulmonary artery systolic pressure; PMR = primary MR; SMR = secondary MR; TAPSE = tricuspid annular plane systolic excursion; TR = tricuspid regurgitation; VC = vena contracta.

**Figure 1:**
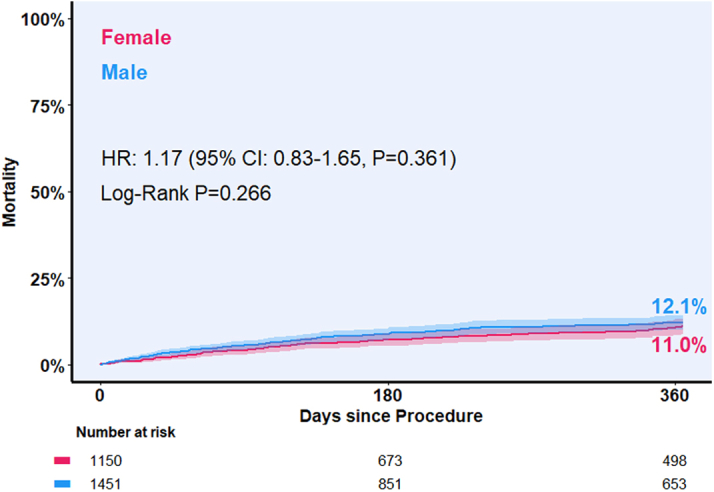
**1-Year All-Cause Mortality According to by Sex** Kaplan-Meier curve showing 1-year all-cause mortality according to sex. One-year all-cause mortality was 11.0% (95% CI: 8.7%-13.2%) in females and 12.1% (95% CI: 10.1%-14.1%) in males (*P* = 0.266).

**Figure 2:**
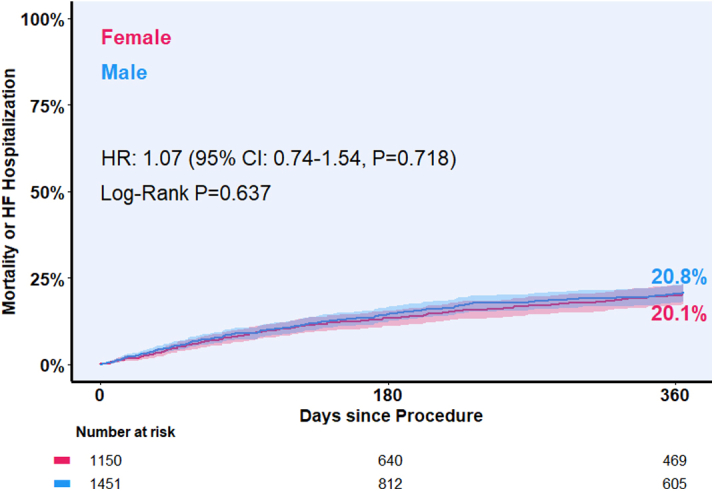
**1-Year All-Cause Mortality or Heart Failure Hospitalization According to Sex** Kaplan-Meier curve depicting 1-year all-cause mortality or heart failure hospitalization according to sex. One-year all-cause mortality or heart failure hospitalization rates were 20.1% (95% CI: 17.2%-22.9%) in females and 20.8% (95% CI: 18.2%-23.3%) in males (*P* = 0.637). HF = heart failure.

**Figure 3:**
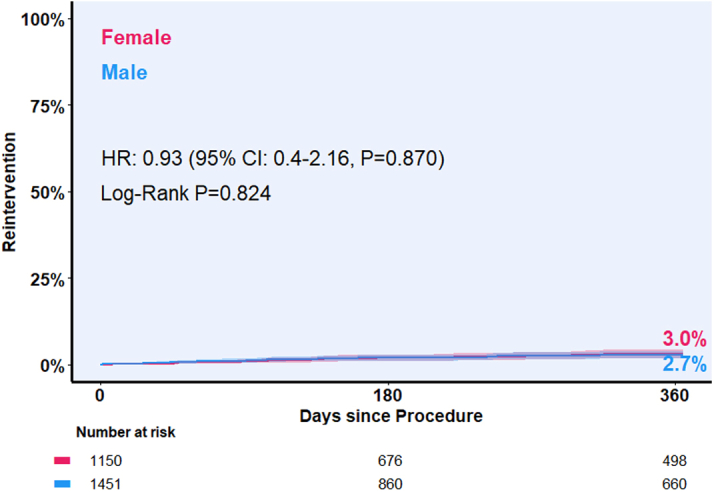
**1-Year Mitral Valve Reintervention Rate According to Sex** Kaplan-Meier curve illustrating the 1-year reintervention rate according to sex. The 1-year mitral valve reintervention rate was 3.1% (95% CI: 1.8%-4.4%) in females and 2.8% (95% CI: 1.7%-3.9%) in males.

**Central Illustration:**
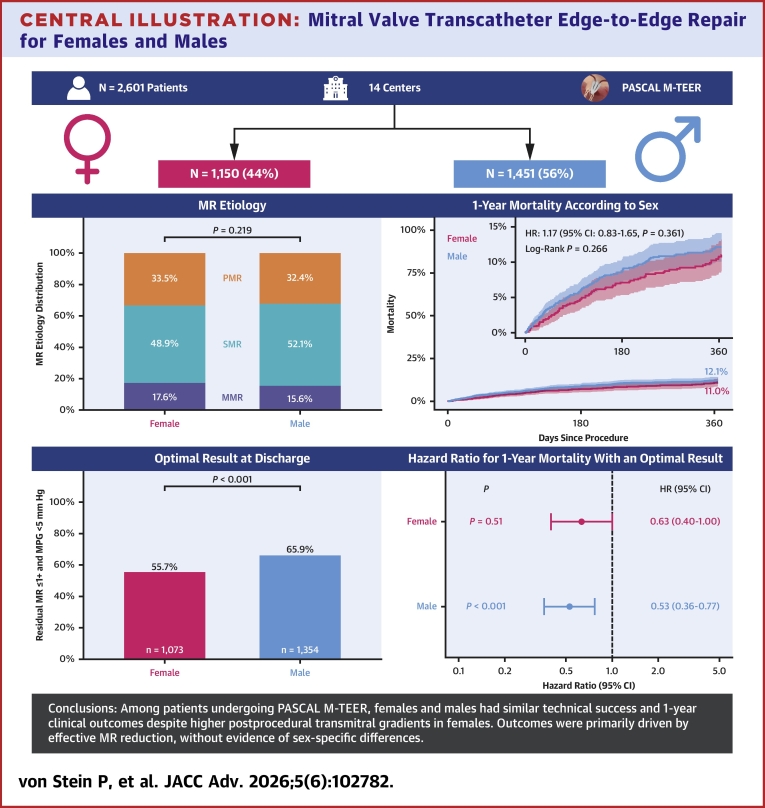
**Mitral Valve Transcatheter Edge-to-Edge Repair for Females and Males** Patients undergoing M-TEER were stratified according to sex. (A) The distribution of MR etiology did not differ between females and males, including PMR, SMR, and MMR. (B) One-year all-cause mortality was similar between females and males. (C) The proportion of patients achieving an optimal result at discharge, defined as residual MR ≤1+ and an MPG <5 mm Hg, significantly differed by sex. (D) Adjusted HRs for 1-year all-cause mortality associated with an optimal result at discharge, stratified by sex. No significant interaction between sex and the association of an optimal result with 1-year all-cause mortality was observed (*P*_interaction_ = 0.609). MMR = mixed MR; MPG = mean mitral valve pressure gradient; MR = mitral regurgitation; M-TEER = mitral transcatheter edge-to-edge repair; PMR = primary MR; REPAIR = REgistry of PAscal for mItral Regurgitation; SMR = secondary MR.

The distribution of MR severity differed significantly between sexes, with a higher prevalence of severe (4+) MR in males (43.5% vs 38.8%; *P* = 0.017) and a higher prevalence of moderate (2+) MR in females (2.3% vs 1.0%; *P* = 0.012). Consistent with these findings, vena contracta width (median 7.0 mm vs 7.8 mm; *P* < 0.001), effective regurgitant orifice area (median 30 mm^2^ vs 39 mm^2^; *P* < 0.001), and regurgitant volume (median 47 mL vs 52 mL; *P* < 0.001) were lower in females. Conversely, at least moderate TR was more prevalent among females (64.7% vs 58.3%; *P* = 0.002). Right ventricular function, assessed by tricuspid valve annular plane excursion, was higher in females (median 19.0 vs 18.0 mm, *P* < 0.001), whereas estimated pulmonary artery systolic pressures were similar between sexes (median 44.0 vs 45.0 mm Hg, *P* = 0.471).

### Procedural outcomes

Procedural outcomes are summarized in Table 3. MVARC technical success rates were similar between females and males (97.0% vs 97.7%; *P* = 0.395). Intraprocedural device detachments were infrequent and similarly distributed (0.5% vs 0.7%; *P* = 0.545), as were aborted procedures (1.8% vs 1.4%; *P* = 0.462).

**Table 3:** Procedural Outcomes

	Females (n = 1,150)	Males (n = 1,451)	*P* Value
Procedural outcomes			
Technical success	1,116/1,150 (97.0)	1,417/1,451 (97.7)	0.395
Intraprocedural device detachment	5/1,089 (0.5)	10/1,363 (0.7)	0.545
Partial detachment	3/1,009 (0.3)	6/1,283 (0.5)	0.739
SLDA	2/1,009 (0.2)	4/1,283 (0.3)	0.700
Procedure aborted	21/1,146 (1.8)	20/1,439 (1.4)	0.462
Procedural death	1/1,150 (0.1)	0/1,451 (0.0)	0.442
Conversion to open heart surgery	0/1,099 (0.0)	2/1,395 (0.1)	0.507
Leaflet damage	2/1,137 (0.2)	5/1,438 (0.3)	0.475
Devices implanted			**<0.001**
1	804/1,150 (69.9)	803/1,451 (55.3)	
2	310/1,150 (27.0)	580/1,451 (40.0)	
3	15/1,150 (1.3)	46/1,451 (3.2)	
4	0/1,150 (0.0)	2/1,451 (0.1)	
Number of devices implanted	1.0 (1.0-2.0) (n = 1,150)	1.0 (1.0-2.0) (n = 1,451)	**<0.001**
P10 only	454/1,150 (39.5)	568/1,451 (39.1)	
Ace only	656/1,150 (57.0)	827/1,451 (57.0)	
Both P10 and Ace	19/1,150 (1.7)	36/1,451 (2.5)	
Independent grasping	333/578 (57.6)	421/758 (55.5)	0.483
Postprocedural 3D-MVOA, cm^2^	2.0 (1.7-2.6) (n = 228)	2.5 (2.0-3.1) (n = 313)	**<0.001**
Procedure duration, minutes	76 (55-101) (n = 989)	80 (59-109) (n = 1,258)	**<0.001**

Values are n/N (%) or median (Q1-Q3). **Bold** values indicate statistical significance.

SLDA = single leaflet device attachment; other abbreviations as in Table 2.

One device was implanted in 69.9% of females vs 55.3% of males, 2 devices in 27.0% vs 40.0%, and at least 3 devices in 1.3% vs 3.3%, respectively (*P* < 0.001). The PASCAL P10 was used in 39.5% of females and 39.1% of males, and the PASCAL Ace in 57.0% of both groups. The combined use of PASCAL P10 and Ace devices occurred in 1.7% of females and 2.5% of males. The median procedure duration was slightly shorter in females (76 minutes vs 80 minutes; *P* < 0.001). In the subset of patients with available data, residual 3D-MVOA was smaller in females (median 2.0 cm^2^ vs 2.5 cm^2^; *P* < 0.001).

### Echocardiographic outcomes

Echocardiographic outcomes are summarized in Table 4. At discharge, an optimal result was achieved less frequently in females than in males (55.7% vs 65.9%; *P* < 0.001) (Central Illustration). Rates of residual MR ≤ 1+ were similar between sexes (71.3% vs 74.6%; *P* = 0.066). MPG was higher in females (median 3.3 vs 3.0 mm Hg; *P* < 0.001), with a higher proportion of females exhibiting a gradient ≥5 mm Hg (24.0% vs 14.0%; *P* < 0.001).

**Table 4:** Echocardiographic and Clinical Outcomes

	Females (n = 1,150)	Males (n = 1,451)	*P* Value
Echocardiographic outcomes			
Echocardiographic outcomes at discharge			
MR ≤2+	1,083/1,115 (97.1)	1,362/1,404 (97.0)	0.952
MR ≤1+	795/1,115 (71.3)	1,048/1,404 (74.6)	0.066
MPG, mm Hg	3.3 (2.8-4.6) (n = 1,074)	3.0 (2.0-4.0) (n = 1,357)	**<0.001**
≥5 mm Hg	258/1,074 (24.0)	190/1,357 (14.0)	**<0.001**
Optimal result	598/1,073 (55.7)	892/1,354 (65.9)	**<0.001**
Echocardiographic outcomes at short-term follow-up^a^			
MR ≤2+	576/610 (94.4)	738/773 (95.5)	0.446
MR ≤1+	405/610 (66.4)	543/773 (70.2)	0.141
MPG, mm Hg	3.5 (3.0-4.8) (n = 586)	3.0 (2.0-4.0) (n = 738)	**<0.001**
≥5 mm Hg	141/586 (24.1)	103/738 (14.0)	**<0.001**
Optimal result	309/586 (52.7)	463/738 (62.7)	**<0.001**
Echocardiographic outcomes at latest follow-up^b^			
MR ≤2+	454/481 (94.4)	575/614 (93.6)	0.703
MR ≤1+	315/481 (65.5)	410/614 (66.8)	0.702
MPG, mm Hg	3.9 (3.0-5.0) (n = 480)	3.0 (2.0-4.0) (n = 608)	**<0.001**
≥5 mm Hg	133/480 (27.7)	81/608 (13.3)	**<0.001**
Optimal result	237/479 (49.5)	363/608 (59.7)	**<0.001**
Clinical outcomes			
NYHA functional class at short-term follow-up^a^			0.144
I	92/611 (15.1)	148/766 (19.3)	
II	295/611 (48.3)	356/766 (46.5)	
III	200/611 (32.7)	241/766 (31.5)	
IV	24/611 (3.9)	21/766 (2.7)	
≤II	387/611 (63.3)	504/766 (65.8)	0.373
NYHA functional class at latest follow-up^b^			**<0.001**
I	57/541 (10.5)	119/682 (17.4)	
II	253/541 (46.8)	318/682 (46.6)	
III	214/541 (39.6)	210/682 (30.8)	
IV	17/541 (3.1)	35/682 (5.1)	
≤II	310/541 (57.3)	437/682 (64.1)	**0.019**

Values are n/N (%) or median (Q1-Q3). **Bold** values indicate statistical significance.

Abbreviations as in Table 2.

a Short-term follow-up refers to the first postdischarge follow-up assessment.

b Latest follow-up refers to the most recent available follow-up assessment.

Short-term echocardiographic follow-up was available in 50.9% of patients (1,324/2,601) at a median of 58 days (Q1–Q3: 35-99 days). At this time point, an optimal result remained less frequent in females than in males (52.7% vs 62.7%, *P* < 0.001), similarly accompanied by higher MPG and a higher proportion of females with gradients ≥5 mm Hg (24.1% vs 14.0%; *P* < 0.001). Rates of residual MR ≤1+ remained similar (66.5% vs 70.2%; *P* = 0.141).

Latest echocardiographic follow-up was available in 41.8% of patients (1,088/2,601) after a median of 391 days (Q1–Q3: 360-652 days), without differences in follow-up duration between sexes. Findings at latest follow-up were consistent with those observed at discharge and at short-term follow-up.

### Clinical outcomes

One-year all-cause mortality was similar between females and males (11.0% vs 12.1%, *P* = 0.266), with 210 deaths overall (Figure 1 and Central Illustration). The 1-year composite endpoint of all-cause mortality or hospitalization for heart failure occurred in 372 patients and was observed in 20.1% of females and 20.8% of males (*P* = 0.637, Figure 2). One-year reintervention rates were also similar between sexes (Figure 3). MR etiology (primary vs secondary vs mixed) did not significantly interact with sex for all-cause mortality (*P*_interaction_ = 0.396), the composite endpoint (*P*_interaction_ = 0.528), or reintervention (*P*_interaction_ = 0.984).

Additional clinical outcomes are summarized in Table 4. NYHA functional class follow-up was available in 52.9% of patients (1,377/2,601) at short-term follow-up and in 47.0% (1,223/2,601) at 1 year. NYHA functional class improved from baseline to short-term follow-up in both sexes (each *P* < 0.001). At short-term follow-up, NYHA functional class ≤II was achieved in 63.3% of females and 65.8% of males (*P* = 0.373). At latest follow-up, NYHA functional class ≤II was more frequently observed in males than in females (64.1% vs 57.3%; *P* = 0.019).

### Residual MR and MPG as outcome predictors across sexes

In the overall cohort, achievement of an optimal result was associated with lower 1-year all-cause mortality (unadjusted HR: 0.57; 95% CI: 0.43-0.77; *P* < 0.001). This association remained significant after adjustment for sex (adjusted HR: 0.53; 95% CI: 0.36-0.77; *P* < 0.001), whereas sex was not independently associated with 1-year mortality (adjusted HR: 0.76; 95% CI: 0.50-1.13; *P* = 0.171). No interaction between an optimal result, sex, and all-cause mortality was observed (*P*_interaction_ = 0.609) (Central Illustration). These results were confirmed in a fully adjusted model (Supplemental Table 2).

In adjusted Cox regression models including sex, residual MR ≤1+, and MPG <5 mm Hg, residual MR ≤1+ was independently associated with lower 1-year mortality (adjusted HR: 0.57; 95% CI: 0.38-0.84; *P* = 0.005). In contrast, MPG <5 mm Hg was not significantly associated with 1-year mortality (adjusted HR: 0.63; 95% CI: 0.40-1.01; *P* = 0.056). Sex remained nonsignificant in this model (adjusted HR: 0.78; 95% CI: 0.40-1.49; *P* = 0.445). Findings were consistent across fully adjusted models (Supplemental Table 3).

No significant interactions were observed between sex and residual MR ≤1+ (*P*_interaction_ = 0.726) or between sex and MPG <5 mm Hg (*p*_interaction_ = 0.624) with respect to 1-year mortality. These findings were confirmed for the 1-year composite endpoint of all-cause mortality or hospitalization for heart failure.

## Discussion

This study represents the largest sex-specific analysis of patients undergoing M-TEER with the PASCAL system. Among 2,601 patients, comparison of females with males revealed the following principal findings: first, clinical and echocardiographic baseline characteristics differed substantially between sexes; second, although MVARC-defined technical success rates were similar across sexes, an optimal result at discharge was achieved less frequently in females, primarily due to higher MPG; third, achievement of an optimal result was associated with lower 1-year mortality and reduced risk of the 1-year composite endpoint of all-cause mortality or heart failure hospitalization, with no evidence of effect modification by sex; fourth, despite differences in baseline characteristics and postprocedural echocardiographic results, 1-year all-cause mortality, the composite endpoint, and mitral valve reintervention rates did not differ between females and males, and sex was not independently associated with adverse clinical outcomes.

### Baseline assessment and surgical risk assessment

Consistent with prior M-TEER studies, females in this analysis were older, more frequently in NYHA functional class III, and exhibited fewer cardiovascular comorbidities, yet had higher EuroSCORE II.^6^^,^^7^^,^^9^^,^^11^^,^^12^ Although several comorbidities were less prevalent in females, EuroSCORE II was higher due to the disproportionate weighting of age, renal dysfunction, NYHA functional class, and female sex within the score algorithm.^22^ Conversely, several comorbidities that were more frequent in males are not directly incorporated into the model. This finding underscores that EuroSCORE II captures surgical risk rather than global disease complexity and may overestimate risk in elderly females with impaired renal function but fewer atherosclerotic comorbidities.

### Symptom burden and referral patterns

The higher prevalence of NYHA functional class III symptoms among females at presentation may reflect under-recognition of symptoms or delayed referral. Treatment in females may be withheld due to age, frailty, patient refusal, or symptom improvement after conservative treatment, as observed in the VHD II EURObservational Research Programme.^23^ However, referral timing and decision-making pathways could not be directly assessed in this registry. Consequently, M-TEER–specific mechanisms underlying delayed intervention in females remain insufficiently characterized and warrant further investigation.

### Sex-specific differences in guideline-directed medical therapy

With respect to medical therapy, females received renin-angiotensin system inhibitors at similar rates and beta-blockers more frequently than males. However, among renin-angiotensin system inhibitors, ARNIs were used less frequently in females. In addition, females received MRAs and SGLT2i less often, resulting in lower rates of at least triple or quadruple GDMT in the overall cohort. These findings were consistent in the SMR subgroup.

When stratified by LVEF-based heart failure categories, these differences were largely explained by the higher prevalence of HFpEF among females. The lower rates of MRAs, at least triple, and quadruple GDMT in females were primarily driven by the higher prevalence of HFpEF among females, in which these therapies are not recommended.^21^ In contrast, ARNIs remained significantly underused in females with both HFrEF and HFmrEF, and SGLT2i were less frequently prescribed in females with HFrEF, indicating sex-specific differences in GDMT independent of LVEF.

Potential explanations include differences in renal function, prevalence of diabetes mellitus, blood pressure, comedications, allergies/intolerances, and the enrollment period spanning 2019 to 2024, partly preceding the 2021 European Society of Cardiology Heart Failure Guidelines and the 2023 focused update.^21^^,^^24^ Nevertheless, these observations highlight sex-specific differences in GDMT optimization, suggesting suboptimal GDMT optimization in females. This is particularly relevant considering current recommendations advocating for simultaneous initiation of quadruple GDMT, at least at low dose.^21^^,^^24^, ^25^, ^26^

### Echocardiographic characteristics

The similar distribution of MR etiologies represents a novel finding, demonstrating that current M-TEER technology with the PASCAL system enables treatment across a broad spectrum of MR pathologies without sex-based selection.

Despite similar MR etiology distribution, females had smaller left ventricular and left atrial dimensions, higher LVEF, and lower MR severity at baseline. Smaller left ventricular dimensions are consistent with prior literature and may primarily reflect differences in body size rather than attenuated chamber remodeling.^6^^,^^7^^,^^9^ Differences in at least moderate concomitant TR are a recognized phenomenon affecting females and are associated with advanced age and atrial fibrillation.^23^^,^^27^^,^^28^ Moreover, an association between atrial fibrillation and TR has been reported in females but not in males.^23^^,^^27^^,^^28^ These findings should increase awareness regarding staged treatment of TR in females.

### Procedural outcomes, optimal result, and prognostic implications

M-TEER with the PASCAL system was associated with high and similar MVARC-defined technical success rates across sexes. The smaller baseline 3D-MVOA in females, although available for only a limited number of patients, might explain the implantation of fewer devices and shorter procedure times, consistent with prior reports.^7^^,^^9^^,^^11^^,^^12^^,^^15^

Despite similar technical success, an optimal result (comprised of residual MR ≤1+ and MPG <5 mm Hg) was achieved less frequently in females, driven by a lower rate of MPG <5 mm Hg. This observation mirrors findings from a prior Society of Thoracic Surgeons / American College of Cardiology Transcatheter Valve Therapy Registry analysis and may reflect smaller baseline and postprocedural 3D-MVOA in females.^11^

Importantly, although achievement of an optimal result was associated with improved 1-year outcomes, the prognostic benefit was primarily driven by MR reduction to ≤1+, without evidence of effect modification by sex. The mean transmitral pressure gradients appeared to have a lesser impact and, again, showed no evidence of effect modification by sex. The prognostic relevance of achieving residual MR ≤ 1+ has been consistently demonstrated across multiple studies.^29^, ^30^, ^31^, ^32^ In contrast, elevated transmitral gradients have not, and may reflect diastolic transmitral flow rather than true iatrogenic stenosis.^29^ Moreover, transmitral gradients are influenced by heart rate, and females generally have slightly higher resting heart rates than males, often by 5 to 10 beats per minute, which might partly explain higher gradients in females.^33^ Consequently, the uniform dichotomous cutoff of 5 mm Hg may represent an oversimplification and could benefit from refinement accounting for sex and heart rate.

Despite differences in baseline characteristics and postprocedural echocardiographic findings, 1-year all-cause mortality, the composite endpoint of mortality or heart failure hospitalization, and mitral valve reintervention rates were similar between females and males. Sex was not independently associated with adverse clinical outcomes, supporting the feasibility and effectiveness of contemporary M-TEER across sexes and MR etiologies.

### Study limitations

This study is subject to the inherent limitations of a registry. First, clinical data were collected at local sites without independent adjudication, precluding reliable assessment of cause-specific mortality. Similarly, echocardiographic assessments were performed by individual centers without review by an independent echocardiographic core laboratory. Second, the mean transmitral pressure gradients are influenced by heart rate, and heart rate–adjusted assessments may more accurately reflect transmitral flow dynamics; however, heart rate data were not available in this registry. Third, follow-up echocardiographic data were available only in a subset of patients, and NYHA functional class was not uniformly collected at all time points. In addition, longer-term outcomes were not assessed. Fourth, longitudinal data on GDMT at discharge and during follow-up were not available. Fifth, although both iterations of the PASCAL implant (PASCAL P10 and Ace) are currently available, the high proportion of PASCAL P10 use in this analysis does not reflect current clinical practice, in which the PASCAL Ace is predominantly used.^17^ Finally, as the study was conducted exclusively in Germany, direct extrapolation to regions with lower procedural volumes should be made with caution.

## Conclusions

In a large real-world cohort undergoing M-TEER with the PASCAL system, females and males achieved similar procedural success, symptomatic improvement, and 1-year clinical outcomes. Although female patients exhibited higher postprocedural MPG and therefore achieved an optimal result less frequently, these differences did not translate into adverse clinical outcomes. The prognostic benefit of MR reduction to ≤1+ was consistent across sexes, supporting equitable use of contemporary M-TEER in females and males.

Perspectives **Competency in Patient Care and Procedural Skills:** Among patients undergoing PASCAL M-TEER for MR, females and males experienced similar rates of technical success, reduction of MR to ≤1+, symptomatic improvement, and 1-year clinical outcomes despite higher MPGs in females.

**TRANSLATIONAL OUTLOOK:** Future studies should refine sex-specific anatomical and hemodynamic thresholds to tailor the definition of technical success. Longer follow-up is needed to understand the midterm to long-term impact on mortality across sexes.

## Funding support and author disclosures

Dr Stolz has received speaker honoraria from 10.13039/100006520Edwards Lifesciences. Dr Mahabadi has received honoraria, lecture fees, and/or grant support from 10.13039/100002429Amgen, Daiichi-Sankyo, 10.13039/100006520Edwards Lifesciences, Novartis, Sanofi, all unrelated to this work; and he is co-founder of Mycor GmbH, a company focusing on AI-based EKG-algorithms. Dr Stein received speaker honoraria from 10.13039/100006520Edwards Lifesciences. Dr Gerçek served as consultant for Edwards Lifesciences and has received a research grant from the Ruhr-University Bochum as Advanced Clinician Scientist. Dr Rassaf has received honoraria, lecture fees, and grant support from 10.13039/100006520Edwards Lifesciences, 10.13039/100004325AstraZeneca, 10.13039/100004326Bayer, 10.13039/100004336Novartis, Berlin Chemie, Daiicho-Sankyo, 10.13039/100001003Boehringer Ingelheim, 10.13039/501100004191Novo Nordisk, Cardiac Dimensions, and 10.13039/100004319Pfizer, all unrelated to this work; and he is co-founder of Bimyo GmbH, a company that develops cardioprotective peptides, co-founder of Mycor GmbH, a company focusing on AI-based EKG-algorithms and co-founder of Yes2NO, developing nitric oxide-based treatments. Dr Lurz has received institutional fees and research grants from Abbott Cardiovascular, 10.13039/100006520Edwards Lifesciences, and 10.13039/100004374Medtronic. Dr Horn has received an unrestricted research grant from 10.13039/100006520Edwards Lifesciences and speaking honoraria from Abbott Cardiovascular. Dr Schindhelm received speaker honoraria and travel expenses from Edwards Lifesciences, MedMile, and Björn Steiger Stiftung. Dr Möllmann received speaker honoraria from Abbott Cardiovascular and Edwards Lifesciences. Dr Baldus received honorarium for consultation by Edwards Lifesciences and was supported by the Deutsche Forschungsgemeinschaft, Bonn, Germany (397484323). Dr Rudolph received research grants and honoraria for consultation from 10.13039/100006520Edwards Lifesciences. Dr Hausleiter received speaker honoraria from and serves as consultant 10.13039/100006520Edwards Lifesciences and TriCares. Dr Pfister received honorarium for consultation by 10.13039/100006520Edwards Lifesciences. Dr Mauri received speaker Honoria and travel compensation by Edwards Lifesciences and was supported by the Deutsche Forschungsgemeinschaft, Bonn, Germany (397484323). All other authors have reported that they have no relationships relevant to the contents of this paper to disclose.
