# Supplementary material for: Sex-Specific Outcomes in Patients Undergoing Mitral Valve Transcatheter Edge-to-Edge Repair: The REPAIR Study
Source: JACC Adv. 2026 May 13;5(6):102782. doi: 10.1016/j.jacadv.2026.102782 (PMC13309300; doi:10.1016/j.jacadv.2026.102782)
Supplement: Supplemental Tables 1-3 [file mmc1.pdf]

SUPPLEMENTAL MATERIAL

Supplemental Table 1. Baseline Medical Therapy in SMR Patients

Medication	Overall		*p*-value
	**Females (N=559)**	**Males (N=748)**	
ACEi, ARB, or ARNI	378/528 (71.6)	537/703 (76.4)	0.066
ARNI	74/435 (17.0)	213/579 (36.8)	**<0.001**
Beta blocker	459/528 (86.9)	593/703 (84.4)	0.235
MRA	234/528 (44.3)	375/703 (53.3)	**0.002**
SGLT2 inhibitor	114/528 (21.6)	235/704 (33.4)	**<0.001**
Loop diuretic	453/528 (85.8)	628/703 (89.3)	0.074
≥Triple GDMT	207/528 (39.2)	355/703 (50.5)	**<0.001**
Quadruple GDMT	60/528 (11.4)	133/702 (18.9)	**<0.001**
	**HFrEF**		
	**Females (N=165)**	**Males (N=417)**	
ACEi, ARB, or ARNI	123/155 (79.4)	313/388 (80.7)	0.819
ARNI	49/129 (38.0)	172/328 (52.4)	**0.007**
Beta blocker	136/155 (87.7)	344/388 (88.7)	0.878
MRA	103/155 (66.5)	250/388 (64.4)	0.729
SGLT2 inhibitor	46/155 (29.7)	162/390 (41.5)	**0.013**
Loop diuretic	126/155 (81.3)	348/388 (89.7)	**0.012**
≥Triple GDMT	94/155 (60.6)	245/388 (63.1)	0.656
Quadruple GDMT	37/155 (23.9)	104/388 (26.8)	0.551
	**HFmrEF**		
	**Females (N=112)**	**Males (N=124)**	
ACEi, ARB, or ARNI	77/107 (72.0)	91/119 (76.5)	0.534
ARNI	9/81 (11.1)	28/95 (29.5)	**0.005**
Beta blocker	97/107 (90.7)	96/119 (80.7)	0.053
MRA	39/107 (36.4)	54/119 (45.4)	0.220
SGLT2 inhibitor	25/107 (23.4)	32/119 (26.9)	0.648
Loop diuretic	97/107 (90.7)	101/119 (84.9)	0.265
≥Triple GDMT	36/107 (33.6)	50/119 (42.0)	0.247
Quadruple GDMT	10/107 (9.3)	11/119 (9.2)	>0.999
	**HFpEF**		
	**Females (N=278)**	**Males (N=197)**	
ACEi, ARB, or ARNI	175/262 (66.8)	127/186 (68.3)	0.819
ARNI	15/222 (6.8)	12/148 (8.1)	0.775
Beta blocker	224/262 (85.5)	146/186 (78.5)	0.072
MRA	90/262 (34.4)	68/186 (36.6)	0.703
SGLT2 inhibitor	43/262 (16.4)	37/185 (20.0)	0.396
Loop diuretic	227/262 (86.6)	170/186 (91.4)	0.158
≥Triple GDMT	76/262 (29.0)	55/186 (29.6)	0.981

Quadruple GDMT	13/262 (5.0)	16/185 (8.6)	0.173
Values are n/N (%). **Bold** values indicate statistical significance. *ACEi = angiotensin-converting enzyme inhibitor; ARB = angiotensin receptor blocker; ARNI = angiotensin receptor neprilysin inhibitor; GDMT = guideline-directed medical therapy; HFmrEF = heart failure with mildly reduced ejection fraction, defined by left ventricular ejection fraction 41-49%; HFpEF = heart failure with preserved ejection fraction, defined by left ventricular ejection fraction ≥50%; HFrEF = heart failure with reduced ejection fraction, defined by left ventricular ejection fraction ≤40%; MR = mitral regurgitation; MRA = mineralocorticoid receptor antagonist; SGLT2 = sodium-glucose cotransporter-2; SMR = secondary MR. Classification into HFrEF, HFmrEF, and HFpEF was based exclusively on left ventricular ejection fraction. Fourteen patients were excluded from the ejection fraction-stratified analysis due to missing data.*			

Supplemental Table 2. Multivariable Cox Regression for 1-Year All-Cause Mortality (Optimal Result)

	Univariable			Multivariable		
	**HR**	**95% CI**	***p*-value**	**HR**	**95% CI**	***p*-value**
Optimal result	0.57	0.43-0.77	**<0.001**	0.63	0.46-0.87	**0.005**
Sex, female vs. male	0.85	0.65-1.12	0.256	0.82	0.58-1.17	0.274
Age, per year	0.95	0.99-1.01	0.950	0.99	0.97-1.01	0.488
NYHA, per class increase	2.34	1.81-3.01	**<0.001**	1.96	1.44-2.68	**<0.001**
Diabetes mellitus	1.29	0.95-1.75	0.102	1.14	0.79-1.63	0.479
Coronary artery disease	1.51	1.13-2.02	**0.006**	1.33	0.91-1.95	0.146
Previous myocardial infarction	1.06	0.75-1.51	0.736	0.91	0.59-1.41	0.671
Previous cardiac surgery	1.56	1.14-2.12	**0.005**	1.29	0.88-1.88	0.195
eGFR, per 10 mL/min	0.87	0.82-0.93	**<0.001**	0.93	0.85-1.00	0.068
LVEF, per 10% increase	0.91	0.83-1.00	0.053	0.94	0.84-1.06	0.341
TR severity, per grade increase	1.44	1.22-1.71	**<0.001**	1.34	1.11-1.63	**0.003**

Bold values indicate statistical significance. *CI = confidence interval; eGFR = estimated glomerular filtration rate (calculated using the Cockcroft–Gault equation); HR = hazard ratio; LVEF = left ventricular ejection fraction; NYHA = New York Heart Association; TR = tricuspid regurgitation.*

Supplemental Table 3. Multivariable Cox Regression for 1-Year All-Cause Mortality (Individual Components of an Optimal Result)

	**Univariable**			**Multivariable**		
	**HR**	**95% CI**	***p*-value**	**HR**	**95% CI**	***p*-value**
Residual MR ≤1+	0.54	0.40-0.72	**<0.001**	0.61	0.44-0.85	**0.003**
MPG <5 mmHg	0.67	0.48-0.94	**0.019**	0.76	0.51-1.11	0.155
Sex, female vs. male	0.85	0.65-1.12	0.256	0.85	0.60-1.22	0.381
Age, per year	0.95	0.99-1.01	0.950	0.99	0.97-1.01	0.394
NYHA, per class increase	2.34	1.81-3.01	**<0.001**	1.92	1.40-2.63	**<0.001**
Diabetes mellitus	1.29	0.95-1.75	0.102	1.16	0.81-1.67	0.429
Coronary artery disease	1.51	1.13-2.02	**0.006**	1.30	0.88-1.91	0.185
Previous myocardial infarction	1.06	0.75-1.51	0.736	0.90	0.57-1.40	0.633
Previous cardiac surgery	1.56	1.14-2.12	**0.005**	1.35	0.92-1.97	0.128
eGFR, per 10 mL/min	0.87	0.82-0.93	**<0.001**	0.93	0.86-1.01	0.075
LVEF, per 10% increase	0.91	0.83-1.00	0.053	0.94	0.83-1.06	0.294
TR severity, per grade increase	1.44	1.22-1.71	**<0.001**	1.37	1.13-1.67	**0.002**

Bold values indicate statistical significance. *CI = confidence interval; eGFR = estimated glomerular filtration rate (calculated using the Cockcroft–Gault equation); HR = hazard ratio; LVEF = left ventricular ejection fraction; MPG = mean mitral valve pressure gradient; MR = mitral regurgitation; NYHA = New York Heart Association; TR = tricuspid regurgitation.*
