# Supplementary material for: Sex-Specific Outcomes in Patients Undergoing Mitral Valve Transcatheter Edge-to-Edge Repair: The REPAIR Study
Source: JACC Adv. 2026 May 13;5(6):102782. doi: 10.1016/j.jacadv.2026.102782 (PMC13309300; doi:10.1016/j.jacadv.2026.102782)
Supplement: Supplemental Appendix [file mmc2.docx]

The REPAIR investigators:

Ralph-Stephan von Bardeleben, MD

Cecilia Ennin, cand. Med

Kai Peter Friedrichs, MD

Christina Grothusen, MD

Malte Kelm, MD

Georg Nickenig, MD

Kerstin Piayda, MD

Tobias Ruf, MD

Leonhard Moritz Schneider, MD

Holger Thiele, MD

Laila Widmann, MD
